# Predicting native kidney volume regression after transplantation in autosomal-dominant polycystic kidney disease

**DOI:** 10.1093/ckj/sfag376

**Published:** 2026-10-22

**Authors:** Yuki Hasegawa, Takahisa Hiramitsu, Hajime Sasaki, Taiki Ogasa, Shigeaki Nakazawa, Masato Shizuku, Kentaro Ide, Yuji Hidaka, Yu Kijima, Hirotake Kodama, Masayuki Tasaki, Keizo Kaku, Shunji Narumi, Takaaki Kobayashi, Yoshihiko Watarai

**Affiliations:** Departments of Transplant Surgery, Transplant Medicine, and Endocrine Surgery, Japanese Red Cross Aichi Medical Center Nagoya Daini Hospital, Nagoya, Japan; Department of Renal Transplant Surgery, Aichi Medical University School of Medicine, Nagakute, Japan; Departments of Transplant Surgery, Transplant Medicine, and Endocrine Surgery, Japanese Red Cross Aichi Medical Center Nagoya Daini Hospital, Nagoya, Japan; Department of Kidney Transplant Surgery, Sapporo City General Hospital, Sapporo, Japan; Department of Urology, Juntendo University Graduate School of Medicine, Tokyo, Japan; Department of Urology, Graduate School of Medicine, The University of Osaka, Osaka, Japan; Department of Renal Transplant Surgery, Aichi Medical University School of Medicine, Nagakute, Japan; Department of Gastroenterological and Transplant Surgery, Graduate School of Biomedical and Health Sciences, Hiroshima University, Hiroshima, Japan; Department of Surgery, Japanese Red Cross Kumamoto Hospital, Kumamoto, Japan; Department of Urology, Tokyo Women’s Medical University, Tokyo, Japan; Department of Urology, Hirosaki University Graduate School of Medicine, Aomori, Japan; Division of Urology, Department of Regenerative and Transplant Medicine, Graduate School of Medical and Dental Sciences, Niigata University, Niigata, Japan; Department of Surgery and Oncology, Graduate School of Medical Sciences, Kyushu University, Fukuoka, Japan; Departments of Transplant Surgery, Transplant Medicine, and Endocrine Surgery, Japanese Red Cross Aichi Medical Center Nagoya Daini Hospital, Nagoya, Japan; Department of Renal Transplant Surgery, Aichi Medical University School of Medicine, Nagakute, Japan; Departments of Transplant Surgery, Transplant Medicine, and Endocrine Surgery, Japanese Red Cross Aichi Medical Center Nagoya Daini Hospital, Nagoya, Japan

**Keywords:** autosomal-dominant polycystic kidney disease, external validation, kidney transplantation, native nephrectomy, prediction model

## Abstract

**Background:**

Native polycystic kidneys often regress after kidney transplantation in patients with autosomal-dominant polycystic kidney disease (ADPKD), but the degree of regression varies widely, and no externally validated preoperative prediction model is available. Therefore, we aimed to develop and externally validate a preoperative model to predict post-transplant native kidney volume changes.

**Methods:**

This multicentre, retrospective study included 224 patients with ADPKD who underwent kidney transplantation at 11 Japanese centres. Patients were divided by centre into non-overlapping development (four centres, *n* = 164) and validation (seven centres, *n* = 60) sets. Total kidney volume (TKV) was measured before and 1 year after transplantation. The TKV change ratio (TKVCR) was calculated as post-transplantation TKV divided by baseline TKV. Multivariable linear regression was used to identify preoperative predictors.

**Results:**

The final model included five preoperative predictors: body mass index, age², log₁₀(TKV), systolic blood pressure, and log₁₀(dialysis duration + 1) (*R*²=0.168). On external validation, the *R*² was 0.255, mean absolute error was 0.122, and root mean squared error was 0.154; however, individual-level prediction was limited. In an exploratory binary classification using the overall cohort mean TKVCR (0.62) as the cutoff, accuracy was 0.783, negative predictive value was 0.862, and the area under the receiver operating characteristic curve was 0.850 (95% confidence interval, 0.743–0.947).

**Conclusions:**

Preoperative factors were associated with post-transplant native kidney volume change, but accuracy for individual patients was limited. These findings provide preliminary evidence that volume changes may be partly predictable.

KEY LEARNING POINTS **What was known:**

- Native polycystic kidneys often regress spontaneously after kidney transplantation, but the degree of regression varies widely between individuals.
- No externally validated preoperative model was available to identify patients likely to experience substantial native kidney volume regression.

**This study adds:**

- A preoperative model incorporating body mass index, age, native kidney volume, systolic blood pressure, and dialysis duration was developed and evaluated in seven centres that were not used for model fitting.
- Explained variation was limited (validation *R*²=0.255) and individual predictions were compressed towards the mean; an exploratory binary classification yielded an area under the receiver operating characteristic curve of 0.850.

**Potential impact:**

- These findings provide preliminary evidence that post-transplant native kidney volume changes may be partly predictable from preoperative information.
- The model cannot currently inform decisions about native nephrectomy; whether predicted volume change relates to symptoms, quality of life or subsequent nephrectomy has not been examined and requires further validation.
- The findings may serve as a starting point for prospective studies examining the relationship with patient-centred outcomes.

## INTRODUCTION

Autosomal-dominant polycystic kidney disease (ADPKD) is an inherited kidney disorder characterized by progressive cystic enlargement and is a common indication for kidney transplantation. Approximately half of patients with ADPKD develop end-stage renal disease by age 60 years, and kidney transplantation is frequently selected as renal replacement therapy [1]. Post-transplantation outcomes in patients with ADPKD are comparable to or more favourable than those in patients with other primary kidney diseases [2].

A unique clinical challenge in ADPKD transplantation is the management of enlarged native polycystic kidneys at the time of surgery. Native nephrectomy is considered for patients with markedly enlarged kidneys that compromise graft placement or cause major compressive symptoms such as abdominal distension, pain, early satiety, or recurrent cyst infection or haemorrhage [3–5]. However, nephrectomy of massively enlarged polycystic kidneys is associated with prolonged operative time, increased blood loss, more perioperative complications, and potential sensitization from blood transfusions [3, 4, 6, 7].

Native polycystic kidneys often regress spontaneously after successful kidney transplantation [8, 9]; however, the degree of regression varies between individuals. Native kidney volume change is an imaging measurement, and its relationship with patient-centred outcomes, such as relief of abdominal symptoms, adequacy of space for graft placement, need for subsequent native nephrectomy, and quality of life, has not been systematically examined.

The decision to perform native nephrectomy is made on an individual basis, considering the kidney size, symptom burden, space for graft placement, perioperative risk, and patient preferences. Information on the expected degree of spontaneous regression post-transplantation may inform this assessment, but no established method exists to estimate it preoperatively. Here, we developed a model predicting native kidney volume change 1-year post-transplantation from preoperative information using retrospective multicentre data from Japan and evaluated it in centres that were not used for model fitting.

## MATERIALS AND METHODS

### Study design and participants

This multicentre, retrospective, observational study was conducted across 11 transplant centres in Japan (Table S1). Adult patients with ADPKD who underwent kidney transplantation at the participating institutions were eligible for inclusion. A total of 295 patients were initially identified. Patients with (i) bilateral native nephrectomy at transplantation; (ii) prior unilateral or bilateral native nephrectomy; (iii) prior intervention for native polycystic kidneys, including transcatheter arterial embolization; (iv) graft loss or death within 1 year; or (v) insufficient imaging data were excluded. Finally, 224 patients were included in the analysis.

The study was approved by the Institutional Review Board of Japanese Red Cross Aichi Medical Center Nagoya Daini Hospital (approval number 1662T) and the review boards of participating centres.

### Imaging analysis and kidney volume measurement

Native polycystic kidney volume was assessed using non-contrast abdominal computed tomography performed pre-transplantation and at 1-year post-transplantation (±3 months). Three-dimensional volumetric analysis was performed using the cyst-kidney analysis module of semi-automated software (SYNAPSE VINCENT^®^, Fujifilm Medical Systems, Tokyo, Japan), whose accuracy and reproducibility for ADPKD kidney volumetry were validated previously [10]. Total kidney volume (TKV) was calculated as the sum of the right and left native kidney volumes, including both cystic and parenchymal components.

Eight investigators performed volumetric analysis. For each scan, automatically extracted kidney contours were visually inspected and manually corrected where necessary. Scans from four centres were measured by a single investigator; at the remaining seven centres, investigators measured the scans from their own institution. No cross-training programme, duplicate measurement, or central adjudication was undertaken for this study. Assessors were not blinded to clinical information or scan time point. In patients in whom only one native kidney was present at 1-year post-transplantation, TKV was derived from that kidney at both time points.

### Variables

Sixteen candidate predictors were specified in advance (Table S2). Because no predictor of post-transplant native kidney volume change has been established in ADPKD, candidates were not narrowed by hypothesis; we specified all recipient, kidney and dialysis-related, cardiovascular, transplant-related, and donor variables that were available pre-transplantation and could be collected uniformly across centres. Post-transplant TKV was used only to derive the outcome. Of these, unilateral native nephrectomy at transplantation changes the basis on which TKV is calculated. It was therefore specified in advance to be examined in a sensitivity analysis (Table S3) rather than entered as a predictor.

### Statistical analysis

#### Predictor selection

The remaining 15 candidates were examined by univariable linear regression; those with *P* < .10 were included in the multivariable model. The final model was obtained by backward elimination based on model fit; in the re-analysis, this was formalized using the Akaike information criterion (AIC).

#### Transformations

Age was entered as age², and TKV and dialysis duration were log₁₀-transformed. Log₁₀(TKV) was used because TKV follows a log-normal distribution in ADPKD, and log-transformed TKV is used as standard in the Mayo classification [11]. Log₁₀(dialysis months + 1) was used because the distribution was concentrated at zero, reflecting the high proportion of pre-emptive transplants (72.6%), with a long right tail. Linearity on the original scales was formally assessed using restricted cubic splines (Fig. S1). A sensitivity analysis using untransformed variables was also performed.

#### Missing data

Missing values were imputed using the median of the full dataset (*n* = 225, before the exclusion of one patient lacking outcome data). Missingness before imputation did not exceed 4.5% (Table S2). A sensitivity analysis using medians derived from the development set alone gave essentially unchanged results (Table S4).

#### Centre allocation

The development and validation sets were allocated at the centre-level, aiming for an approximate 70:30 split of patients while keeping the number of contributing centres broadly comparable. Because one centre contributed 115 of the 224 patients (51.3%), any allocation approximating this ratio required that centre to be assigned to the development set. The allocation was not randomized and was not based on clinical characteristics of the centres. It was fixed before model estimation and before any assessment of performance. Centre-level sample sizes and TKV change ratio (TKVCR) distributions are shown in Table S5.

#### Internal validation

Optimism in the development set was corrected using bootstrap resampling (1000 replications). In the re-analysis, predictors were held fixed within each bootstrap sample and only the coefficients were re-estimated. As a sensitivity analysis, univariable screening and backward selection were repeated within each bootstrap sample. The predictor transformations were held fixed in both analyses, and imputation was performed before resampling and not repeated within bootstrap samples; the resulting estimates therefore do not capture uncertainty from these steps.

#### Binary classification

As a secondary, exploratory analysis, TKVCR was dichotomized at 0.62. This value corresponds to the mean TKVCR of the overall cohort and was therefore not independent of the validation set. A sensitivity analysis using the mean derived from the development set alone (0.6124) is presented alongside the primary cutoff in Table S6. Because classification performance varied across thresholds, all binary analyses are regarded as exploratory.

#### Performance and uncertainty

External validation was assessed using *R*² (calculated as 1 − SSE/SST, where SSE is the sum of squared errors and SST is the total sum of squares), mean absolute error, root mean squared error, and calibration [12, 13], defined as the intercept and slope obtained by regressing observed TKVCR on predicted TKVCR. Uncertainty in the validation set was estimated separately from the development-set bootstrap: with the fitted model held fixed, patients were resampled with replacement over 2000 replications and percentile intervals were derived. To examine the influence of within-centre correlation, centres rather than patients were resampled in a separate bootstrap analysis (Table S7). This study is reported in accordance with the TRIPOD statement [14].

#### Software and use of artificial intelligence

Statistical analyses were originally performed using EZR (version 1.68) and R (version 4.3.2) [15]. Subsequent confirmation analyses were independently reproduced and verified in R (version 4.6.0) and in Python (version 3.12, statsmodels 0.14), with agreement confirmed across both implementations. Statistical significance was defined as *P* < .05 (two-sided).

No artificial intelligence tools were used in the original design, data collection, or analysis. In preparing this revision, a large language model (Claude Opus 5, Anthropic) was used to generate analysis code in Python, reproduce and verify the reported results, and perform additional analyses. ChatGPT (GPT-5.6 Sol, OpenAI) was used to review the manuscript and suggest language edits. All analyses were independently validated in R and confirmed by the authors, who take full responsibility for the results and their interpretation.

## RESULTS

### Patient characteristics

A total of 224 patients were included in the final analysis, comprising 164 and 60 patients in the development and validation sets, respectively. Table 1 summarizes the baseline characteristics of the two cohorts. Recipient age, sex, BMI, blood pressure, donor age, and unilateral native nephrectomy rates were comparable between cohorts. However, dialysis duration was longer, and the proportion of pre-emptive transplantation was lower in the validation set. Pre-transplantation TKV tended to be smaller in the development set than in the validation set, although the difference was not statistically significant (*P* = .198). Centre-level sample sizes and TKVCR distributions are shown in Table S5.

**Table 1:** Baseline characteristics of the development and validation cohorts.

Variable	Total cohort *n* = 224	Development set *n* = 164	Validation set *n* = 60	*P*-value
Recipient age (years)	52.0 ± 11.1	51.6 ± 11.1	53.2 ± 10.7	.356
Recipient sex (male/female)	138/86	100/64	38/22	.748
BMI (kg/m²)	22.3 ± 3.1	22.4 ± 3.1	21.9 ± 2.9	.263
Dialysis duration (months)	0 [0.0–9.9]	0.0 [0.0–2.3]	2.5 [0.0–19.0]	<.001
Pre-emptive transplantation, *n* (%)	143 (63.8)	119 (72.6)	24 (40.0)	<.001
Pre-transplant SBP (mmHg)	132.3 ± 16.2	132.4 ± 16.1	131.9 ± 16.7	.827
Pre-transplant DBP (mmHg)	82.1 ± 11.1	81.7 ± 11.1	83.3 ± 11.0	.331
Pre-transplant TKV (mL)	2933 [2000–4046]	2820 [2002–3817]	3228 [1930–5041]	.198
Diabetes mellitus, *n* (%)	13 (5.8)	10 (6.1)	3 (5.0)	1
ABO incompatible, *n* (%)	80 (35.7)	58 (35.4)	22 (36.7)	.858
Pre-transplant DSA positive, *n* (%)	29 (12.9)	25 (15.2)	4 (6.7)	.091
Tolvaptan use, *n* (%)	64 (28.6)	52 (31.7)	12 (20.0)	.087
Donor age (years)	60.5 ± 10.3	60.4 ± 9.9	60.6 ± 11.3	.926
Donor sex (male/female)	92/132	71/93	21/39	.266
Donor serum creatinine (mg/dl)	0.72 ± 0.15	0.73 ± 0.16	0.69 ± 0.13	.152
Unilateral nephrectomy at transplant, *n* (%)	60 (26.8)	41 (25.0)	19 (31.7)	.321

Note: Baseline demographic, clinical, and imaging characteristics of the study participants stratified by development (*n* = 164) and validation (*n* = 60) sets. Data are presented as mean ± standard deviation, median [interquartile range], or number (percentage) as appropriate. Sets were allocated at the centre level; centre-level sample sizes and TKVCR distributions are shown in Table S5.

Abbreviations: BMI, body mass index; DBP, diastolic blood pressure; DSA, donor-specific antibody; SBP, systolic blood pressure; TKV, total kidney volume; TKVCR, total kidney volume change ratio.

### Changes in native polycystic kidney volume post-transplantation

In the overall cohort (*n* = 224), the mean TKVCR at 1-year post-transplantation was 0.621 ± 0.158 (range, 0.205–1.035), corresponding to an average 38% reduction in native kidney volume (Fig. 1). Nineteen patients (8.5%) had a TKVCR of ≥0.85, indicating minimal reduction or a slight increase in native kidney volume. Among the 224 patients, 60 had only one native kidney present at 1-year post-transplantation. TKVCR did not differ between these patients and those with both native kidneys (0.637 versus 0.615, *P* = .37).

**Figure 1:**
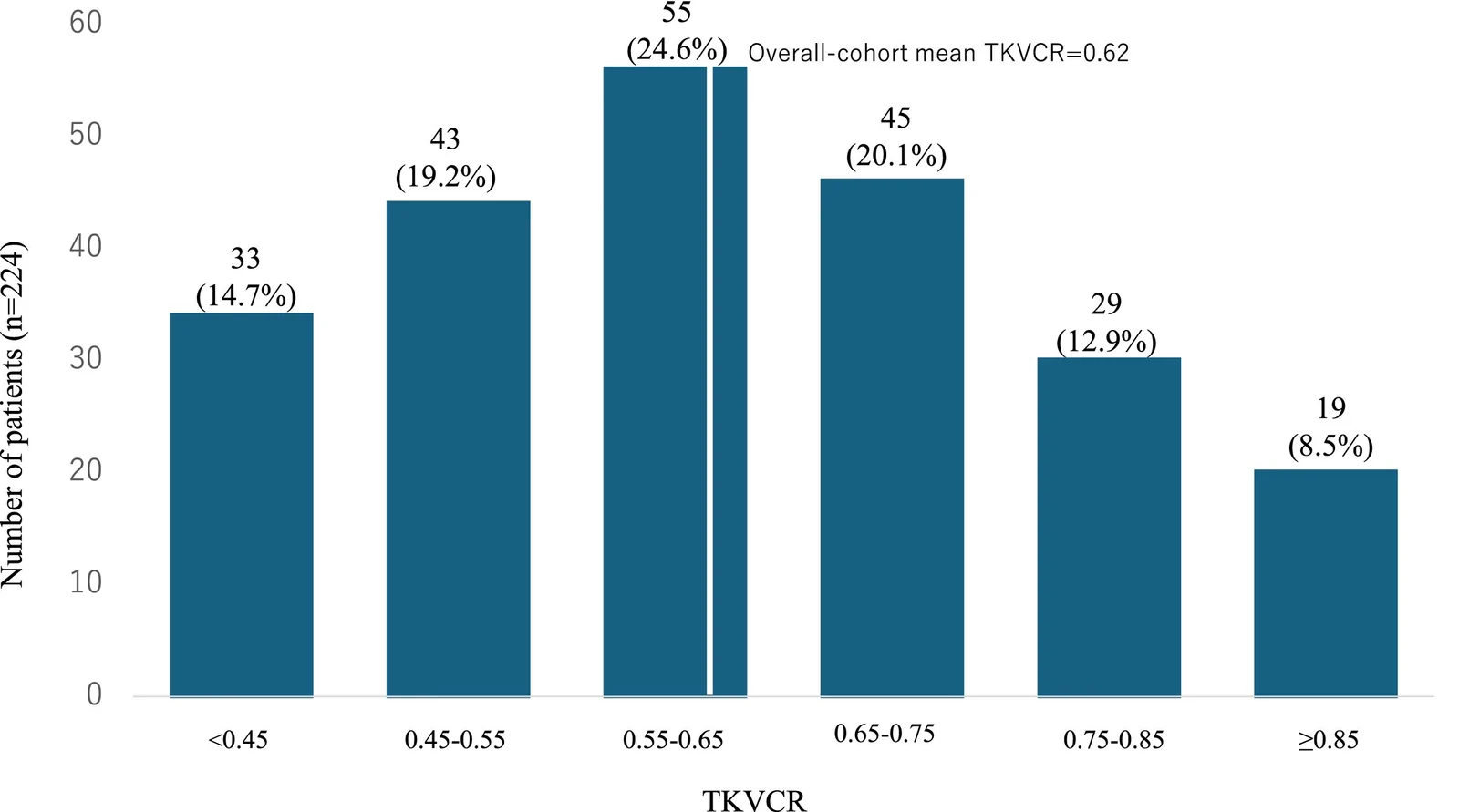
Distribution of TKVCR 1 year after kidney transplantation. Histogram showing the distribution of TKVCR in 224 patients with ADPKD. TKVCR was categorized into six intervals: <0.45 (*n* = 33, 14.7%), 0.45–0.55 (*n* = 43, 19.2%), 0.55–0.65 (*n* = 55, 24.6%), 0.65–0.75 (*n* = 45, 20.1%), 0.75–0.85 (*n* = 29, 12.9%), and ≥0.85 (*n* = 19, 8.5%). The vertical line indicates the mean TKVCR of the overall cohort (0.62), which was used as the threshold in the exploratory binary classification analysis. TKVCR values of 0.62 or greater but below 1.00 still represent a reduction in native kidney volume; values >1.00 indicate an increase. Abbreviation: TKVCR, total kidney volume change ratio.

### Prediction model development

Of the 15 candidate predictors examined, seven met the univariable screening criterion of *P* < .10: pre-transplantation BMI, recipient age, dialysis duration, pre-emptive transplantation, pre-transplantation TKV, systolic blood pressure, and male sex. Backward elimination based on the AIC removed pre-emptive transplantation and male sex, leaving five predictors in the final model (Table 2). Adding male sex did not improve model fit (adjusted *R*²=0.142 without versus 0.138 with male sex; AIC, −176.5 versus −174.8). The derived equation was predicted TKVCR = 0.102 + 0.0125 × BMI + 0.0000238 × age² + 0.0929 × log₁₀(pre-transplant TKV) − 0.00125 × SBP + 0.0257 × log₁₀(dialysis duration [months] + 1).

**Table 2:** Multivariable linear regression model for predicting TKVCR (development set, *n* = 164).

Variable	*β*	95% CI	*P*-value
Pre-transplant BMI	0.0125	0.0055–0.0196	<.001
Recipient age²	2.38 × 10^−5^	2.7 × 10^−6^–4.5 × 10^−5^	.027
log₁₀(pre-transplant TKV)	0.0929	−0.0040 to 0.190	.060
Pre-transplant SBP	−0.00125	−0.0026 to 0.000107	.071
log₁₀(dialysis months + 1)	0.0257	−0.0077 to 0.059	.131

Note: Data are displayed as regression coefficients (*β*) with 95% confidence intervals from the multivariable linear regression model.

Age was modelled as age²; TKV and dialysis duration were log_10_-transformed (log_10_(TKV) and log_10_(months + 1)).

Abbreviations: BMI, body mass index; CI, confidence interval; SBP, systolic blood pressure; TKV, total kidney volume; TKVCR, total kidney volume change ratio.

Assessment using restricted cubic splines showed no clear departure from linearity on the original scales for age or TKV (Fig. S1). The contribution of the transformations to model fit was small (ΔAIC, 1.45), and a sensitivity analysis using untransformed variables yielded similar results (Fig. S1).

Model performance in the development set was *R*²=0.168 (adjusted *R*²=0.142). Bootstrap internal validation yielded an optimism-corrected *R*² of 0.110, with a calibration slope of 0.908 and an intercept of 0.057. When univariable screening and backward selection were repeated within each bootstrap sample, the optimism-corrected *R*² was 0.093.

### External validation and binary classification performance

When applied to the validation set, *R*² (calculated as 1 − SSE/SST) was 0.255, the mean absolute error was 0.122, and the root mean squared error was 0.154. Corresponding 95% confidence intervals (CIs) are shown in Table S7.

The calibration intercept was −0.396 and the slope was 1.671 (95% CI, 1.111–2.222). A slope >1 indicates that observed differences between patients were larger than those represented by the model’s predictions (Fig. 2). The standard deviation of predicted values was 0.061, compared with 0.180 for the observed values. Together with the limited explained variation, this indicates limited accuracy for predicting absolute values in individual patients. A centre-level bootstrap yielded wider intervals for *R*² and the calibration estimates, but not for all performance measures (Table S7).

**Figure 2:**
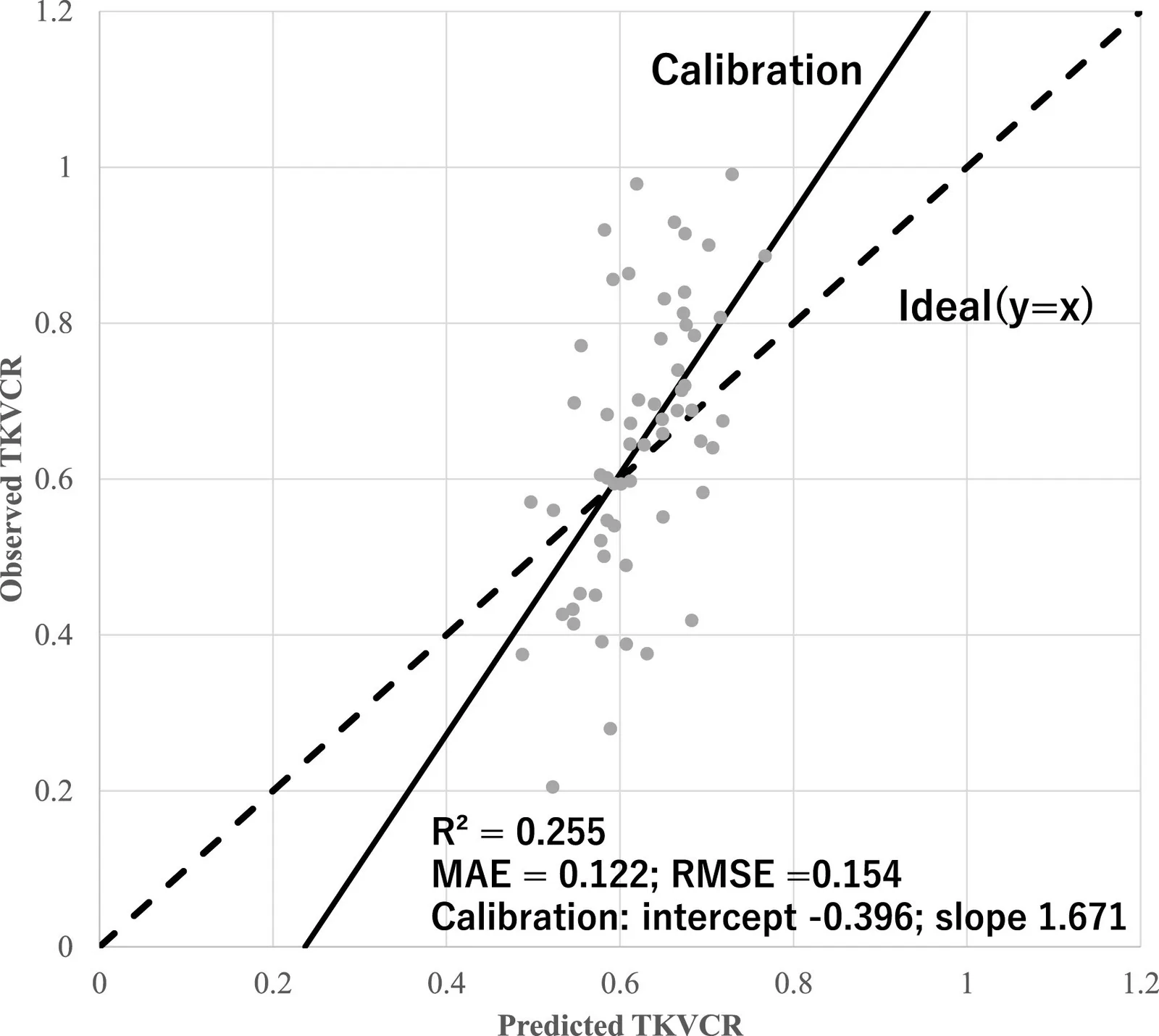
Calibration of the prediction model in the external validation cohort (*n* = 60). Observed TKVCR plotted against predicted TKVCR for the 60 patients in the validation cohort. The dashed line indicates ideal calibration (*y* = *x*) and the solid line is the calibration regression of observed on predicted TKVCR (intercept −0.396; slope 1.671). A slope >1 indicates that observed differences between patients were larger than those represented by the predictions. Abbreviations: MAE, mean absolute error; RMSE, root mean squared error; TKVCR, total kidney volume change ratio.

The higher validation-set *R*² does not indicate improved performance and may reflect sampling variability or case-mix differences; TKVCR variance was 1.46-fold greater (standard deviation, 0.180 versus 0.149).

In the exploratory binary classification at a TKVCR cutoff of 0.62, the accuracy was 0.783, sensitivity was 0.846, specificity was 0.735, positive predictive value was 0.710, negative predictive value (NPV) was 0.862, and Cohen’s *κ* was 0.569 (Table 3 and Fig. 3). The area under the receiver operating characteristic curve (AUC) was 0.850 (95% CI, 0.743–0.947). Using the cutoff derived from the development set alone (0.6124), no validation set observation fell between the two values. The observed classes were therefore identical, and classification differed for a single patient (accuracy, 0.800, AUC, 0.850; Table S6). In the exploratory binary analysis restricted to validation set patients with both native kidneys (*n* = 41), classification performance was similar (AUC, 0.865, NPV, 0.870; Table S3). The AUC reflects the ability to rank patients and does not indicate that individual TKVCR predictions are well calibrated or that classification at this threshold is clinically reliable.

**Figure 3:**
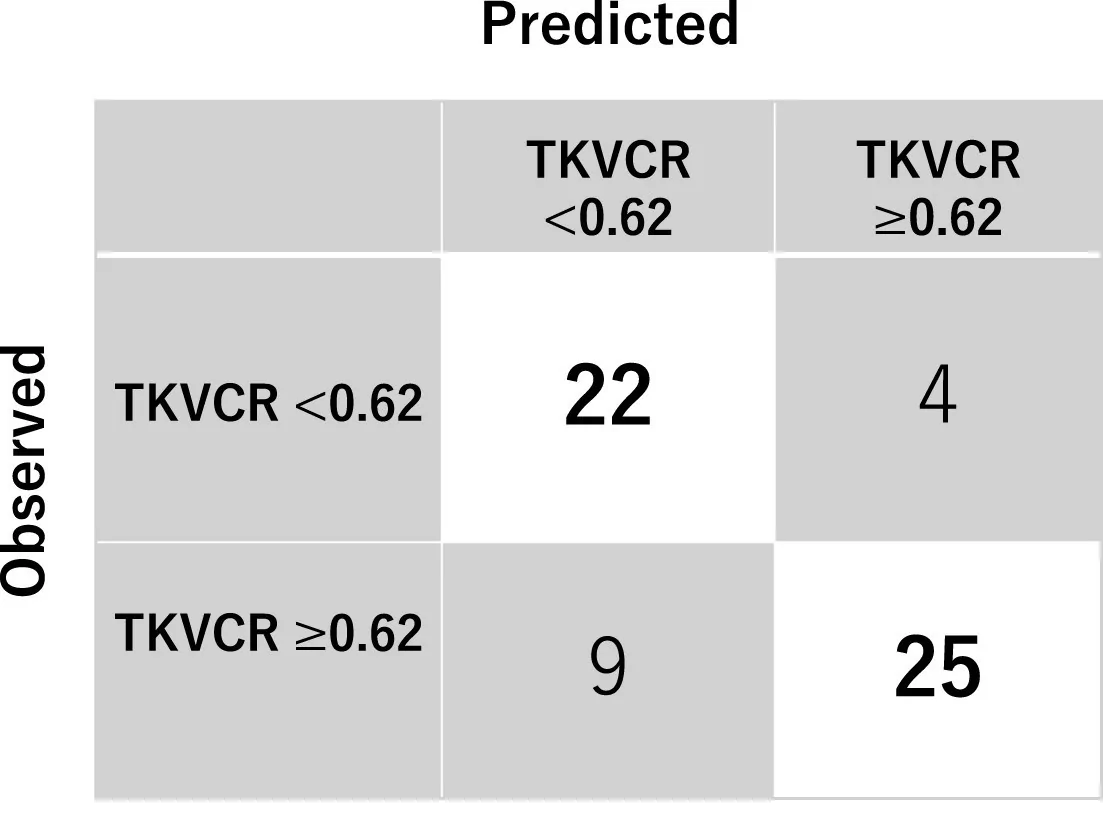
Confusion matrix for the exploratory binary classification in the external validation cohort (*n* = 60). Patients were classified according to whether their TKVCR was below or at/above 0.62, the mean TKVCR of the overall cohort. Accuracy was 0.783, sensitivity 0.846, specificity 0.735, positive predictive value 0.710, and NPV 0.862; the AUC was 0.850 (95% CI 0.743–0.947). This analysis was exploratory, and classification performance varied across alternative thresholds (Table S6). Abbreviation: TKVCR, total kidney volume change ratio.

**Table 3:** Exploratory binary classification performance of the prediction model in the validation cohort.

Metric	Value	95% CI
Accuracy	0.783	0.683–0.883
Sensitivity	0.846	0.697–0.966
Specificity	0.735	0.588–0.885
PPV	0.710	0.543–0.871
NPV	0.862	0.724–0.969
Cohen’s *κ*	0.569	0.356–0.767
AUC	0.850	0.743–0.947

Note: Binary classification in the validation cohort (*n* = 60), dichotomizing the total kidney volume change ratio (TKVCR) at 0.62, the mean TKVCR of the overall cohort. Values are point estimates with 95% confidence intervals obtained by patient-level bootstrap (2000 replications, percentile method), as in Table S7. This threshold was not independent of the validation set, and classification performance varied across alternative thresholds (Table S6); these analyses are therefore exploratory. The AUC reflects the ability to rank patients and does not indicate that individual TKVCR predictions are well calibrated or that classification at this threshold is clinically reliable.

Abbreviations: AUC, area under the receiver operating characteristic curve; CI, confidence interval; NPV, negative predictive value; PPV, positive predictive value; TKVCR, total kidney volume change ratio.

## DISCUSSION

In this multicentre study, we developed and evaluated a preoperative prediction model for native kidney volume change after kidney transplantation in patients with ADPKD. A patient-centred endpoint such as symptom burden, quality of life, or the need for subsequent native nephrectomy would have been preferable. However, only eight of the 224 patients underwent post-transplant native nephrectomy, of whom only one underwent nephrectomy for compressive symptoms; this number was insufficient to support such an endpoint. Volume change was therefore used as a pragmatic surrogate, on the premise that substantial volume reduction may correlate with symptomatic relief; this hypothesis is consistent with the known relationship between kidney size and compressive symptoms in ADPKD, but prospective validation is required.

A model derived from 164 patients was applied to 60 patients from seven centres that were not used for model fitting. Explained variation was limited (*R*² = 0.255), and the calibration slope (1.671) indicated that observed differences between patients were larger than those represented by the predictions. Individual prediction accuracy was therefore limited. In the exploratory binary analysis, the AUC (0.850) indicated discrimination between patients with observed TKVCR <0.62 and ≥0.62 but did not establish that individual predictions are well calibrated or that classification at any threshold is clinically reliable [16]. Recalibration was not attempted, as the validation cohort was too small to reliably support it.

The mean reduction observed (TKVCR, 0.62, ~38%) was consistent with previous findings, including our earlier report of a 37.7% decrease at 1 year [9]. The mechanisms underlying this decrease have not been fully elucidated. After restoration of graft function, native renal blood flow may decrease, leading to progressive atrophy [17–19], while the decline in tubular function may attenuate the cyclic AMP-dependent stimuli that drive cyst growth [20, 21]. Improvement in the uraemic and endocrine milieu, including vasopressin signalling, may further reduce cyst expansion. However, these mechanisms remain speculative.

Higher BMI was associated with less regression, consistent with the association between visceral adiposity, chronic low-grade inflammation [22], and ADPKD progression [23], which may promote remodelling and fibrosis and reduce the reversibility of cystic architecture. In older patients, prolonged cyst expansion may produce cumulative structural change such as cyst wall fibrosis, calcification, and adhesions after prior haemorrhage or infection. Larger pre-transplant TKVs may likewise reflect greater structural burden, as progressive cyst expansion is accompanied by interstitial inflammation and fibrosis [24, 25]. Longer dialysis duration may be a marker of cumulative uraemic and inflammatory exposure as chronic inflammation in dialysis populations [26, 27] and uraemic toxins such as indoxyl sulphate and p-cresyl sulphate activate pro-fibrotic pathways [28]. Meanwhile, higher systolic blood pressure was associated with greater reduction, possibly because post-transplant normalization of blood pressure and volume status reduces native kidney perfusion and facilitates cyst collapse. Volume reduction may thus depend on the interplay between the reversibility of structural change and post-transplant haemodynamic, fluid, and endocrine shifts. The associations of TKV, systolic blood pressure, and dialysis duration with TKVCR were imprecise, with CIs including zero; these interpretations are inferred only from the direction of the observed associations.

No clear consensus has been established regarding the optimal indication for, or timing of, native nephrectomy in ADPKD [29, 30]; nephrectomy should not be performed uniformly [3–6]. Information on the expected degree of spontaneous volume reduction post-transplantation may inform this assessment. However, our findings do not show that predicted volume change is associated with the clinical considerations relevant to that decision, such as symptom burden, feasibility of graft placement, or the subsequent need for nephrectomy. The present findings constitute preliminary evidence that volume change may be partly predictable, rather than evidence that the model can inform clinical decisions.

This study has some limitations. The retrospective design precludes causal inference, and the cohort included only Japanese patients, with a high proportion of pre-emptive and living donor transplants, which may limit generalizability. The model was developed and evaluated in the overall eligible transplant cohort; its performance in patients documented to face a decision about native nephrectomy has not been established. Additionally, external evaluation was based on a single, non-overlapping split at the centre-level, which does not establish the stability of performance across centres. TKVCR distributions differed between centres (Table S5), and centre-level bootstrap intervals for *R*² and calibration were wider (Table S7); with only seven validation centres, these intervals are imprecise.

Furthermore, predictors were selected by a data-driven procedure, and univariable screening may miss predictors that are useful only in combination. Volumetry was derived from routine-care non-contrast computed tomography. No cross-training programme, duplicate measurement, or central adjudication was undertaken; inter-rater reproducibility was not evaluated. As volume was assessed only at 1 year, longer-term patterns could not be examined. Inter-centre differences in surgical and post-transplant management were also not adjusted for, and post-transplantation factors such as calcineurin inhibitor-induced vasoconstriction, immunosuppressive regimen, and graft function recovery were not captured. Sixty recipients had only one native kidney present at 1 year; similar exploratory binary performance in the subgroup with both native kidneys (Table S3) could not fully establish comparability of the continuous outcome. Polycystic liver disease was present in some patients, but its impact was not evaluated. The primary outcome was a volumetric surrogate, and its association with patient-reported outcomes was not evaluated. Future studies should prospectively validate and update the model in larger, more diverse cohorts incorporating clinically relevant outcomes.

In conclusion, we developed a preoperative model incorporating BMI, age, native kidney volume, systolic blood pressure, and dialysis duration, and evaluated it in centres that were not used for model fitting. Preoperative factors were associated with post-transplant native kidney volume change, but accuracy for individual patients was limited. These findings provide preliminary evidence that volume change may be partly predictable and may serve as a starting point for prospective studies examining the relationship between predicted volume change and patient-centred outcomes.

## Supplementary Material

sfag376_Supplemental_File

## Data Availability

The data that support the findings of this study are available from the corresponding author upon reasonable request. Due to privacy and ethical restrictions, individual patient data cannot be publicly shared. Aggregated or anonymized data may be shared with appropriate institutional review board approval and a formal data sharing agreement.
