# Supplementary material for: Predicting native kidney volume regression after transplantation in autosomal-dominant polycystic kidney disease
Source: Clin Kidney J. 2026 Oct 22;19(11):sfag376. doi: 10.1093/ckj/sfag376 (PMC13748871; doi:10.1093/ckj/sfag376)
Supplement: Supplementary file 1 [file sfag376_supplemental_file.docx]

**Supplementary Information**

**Table S1. List of participating institutions and research collaborators**

| Name (Given Family) | Affiliations |
| --- | --- |
| Manabu Okada | Departments of Transplant Surgery, Transplant Medicine, and Endocrine Surgery, Japanese Red Cross Aichi Medical Center Nagoya Daini Hospital, Nagoya, Japan |
| Kenta Futamura |  |
| Tomoki Himeno |  |
| Yuki Shimamoto |  |
| Ayano Ienaga |  |
| Kohei Ishiyama | Department of Renal Transplant Surgery, Aichi Medical University School of Medicine, Aichi, Japan |
| Satoshi Ashimine |  |
| Ken Kawata |  |
| Tatsu Tanabe | Department of Kidney Transplant Surgery, Sapporo City General Hospital, Hokkaido, Japan |
| Yuki Nakagawa | Department of Urology, Juntendo University Graduate School of Medicine, Tokyo, Japan |
| Haruna Kawano |  |
| Tomoki Kimura |  |
| Shuichiro Narita |  |
| Masataka Sano |  |
| Hironori Ohta |  |
| Yoichi Kakuta | Department of Urology, Graduate School of Medicine, The University of Osaka, Osaka, Japan |
| Hiroyuki Tahara | Department of Gastroenterological and Transplant Surgery, Graduate School of Biomedical and Health Sciences, Hiroshima University, Hiroshima, Japan |
| Masahiro Ohira |  |
| Seiichi Shimizu |  |
| Yuki Imaoka |  |
| Shigeyoshi Yamanaga | Department of Surgery, Japanese Red Cross Kumamoto Hospital, Kumamoto, Japan |
| Hideki Ishida | Department of Organ Transplant Medicine, Tokyo Women's Medical University, Tokyo, Japan |
| Shingo Hatakeyama | Department of Urology, Hirosaki University Graduate School of Medicine, Aomori, Japan |
| Tomoko Hamaya |  |
| Shinsuke Kubo | Department of Surgery and Oncology, Graduate School of Medical Sciences, Kyushu University, Fukuoka, Japan |
| Akari Kaba |  |

This multicentre study included 11 institutions across Japan. The table lists the participating institutions and research team members who contributed to patient recruitment, data collection, and study coordination. One institution (Niigata University, represented by Dr. Masayuki Tasaki) contributed to the study with a single investigator, who is listed among the study authors.

**Table S2. Missing values among candidate predictors**

|  | Missing (n) | Missing (%) |
| --- | --- | --- |
| Recipient age | 0 | 0 |
| Recipient sex | 0 | 0 |
| Recipient BMI | 1 | 0.4 |
| Dialysis duration | 0 | 0 |
| Pre-emptive transplantation | 0 | 0 |
| Pre-transplant SBP | 10 | 4.5 |
| Pre-transplant DBP | 10 | 4.5 |
| Pre-transplant TKV | 0 | 0 |
| History of diabetes mellitus | 0 | 0 |
| ABO incompatible | 0 | 0 |
| Pre-transplant DSA positive | 0 | 0 |
| History of tolvaptan use | 0 | 0 |
| Donor age | 0 | 0 |
| Donor sex | 0 | 0 |
| Donor serum creatinine | 3 | 1.3 |
| Unilateral nephrectomy at transplant | 0 | 0 |

Missing values are shown as n (%), with the analysis cohort as the denominator (n=224). These 16 variables constituted the candidate predictors specified in advance. Missingness was low overall (maximum 4.5%). Missing values were distributed between the development and validation sets as follows: BMI, 1 and 0; systolic and diastolic blood pressure, 4 and 6; donor serum creatinine, 2 and 1, respectively.

**Abbreviations:** BMI, body mass index; DBP, diastolic blood pressure; DSA, donor-specific antibody; SBP, systolic blood pressure; TKV, total kidney volume.

**Table S3. Sensitivity analysis: Model performance in validation set patients without concomitant unilateral native nephrectomy**

| **Analysis** | **n** | **Accuracy** | **Sensitivity** | **Specificity** | **PPV** | **NPV** | **AUC** |
| --- | --- | --- | --- | --- | --- | --- | --- |
| **Primary analysis (all validation patients)** | **60** | **0.783** | **0.846** | **0.735** | **0.710** | **0.862** | **0.850** |
| Sensitivity analysis (bilateral kidneys only) | 41 | 0.805 | 0.812 | 0.800 | 0.722 | 0.870 | 0.865 |

The prediction equation derived from the full development set (n=164) was applied to the full validation set (n=60; primary analysis) and to the subset of validation set patients who did not undergo concomitant unilateral native nephrectomy at transplantation (n=41; sensitivity analysis). Binary classification used TKVCR=0.62 as the cutoff.

**Abbreviations:** AUC, area under the receiver operating characteristic curve; NPV, negative predictive value; PPV, positive predictive value; TKVCR, total kidney volume change ratio.

(A)

TKVCR

| dialysis duration (months) | 変化率 |
| --- | --- |
| 0 | 0.494481 |
| 0 | 0.574142 |
| 10.5 | 0.588471 |
| 0 | 0.500571 |
| 7 | 0.465647 |
| 0 | 0.756224 |
| 0 | 0.750648 |
| 0 | 0.479551 |
| 74.2333 | 0.59425 |
| 0 | 0.893328 |
| 0 | 0.500837 |
| 0 | 0.603638 |
| 236.9 | 0.70117 |
| 0 | 0.698152 |
| 9.83333 | 0.767467 |
| 0 | 0.562812 |
| 27.0333 | 0.859524 |
| 9.3 | 0.883616 |
| 0 | 0.725572 |
| 0 | 0.736994 |
| 0 | 0.837218 |
| 0 | 0.818142 |
| 0 | 0.520152 |
| 0 | 0.734597 |
| 0 | 0.933829 |
| 0 | 0.586142 |
| 0 | 0.528263 |
| 0 | 0.626274 |
| 13.9333 | 0.839912 |
| 0 | 0.792157 |
| 0 | 0.631642 |
| 0 | 0.813317 |
| 0 | 0.781682 |
| 2.3 | 0.833428 |
| 0 | 0.764882 |
| 0 | 0.572269 |
| 0 | 0.481976 |
| 0 | 0.739567 |
| 0 | 0.771493 |
| 0 | 0.559118 |
| 0 | 0.488235 |
| 0 | 0.61084 |
| 0.3 | 0.574447 |
| 0 | 0.68163 |
| 2.4 | 0.584781 |
| 0 | 0.746102 |
| 0 | 0.563294 |
| 164.133 | 0.420662 |
| 0 | 0.531075 |
| 38 | 0.617647 |
| 0 | 0.509816 |
| 39 | 0.6555 |
| 0 | 0.452134 |
| 0 | 0.945797 |
| 0 | 0.537952 |
| 0 | 0.527 |
| 0 | 0.555858 |
| 0 | 0.577286 |
| 0 | 0.563244 |
| 5 | 0.399003 |
| 0 | 0.54137 |
| 6 | 0.729573 |
| 0 | 0.400181 |
| 0 | 0.839955 |
| 0 | 0.670281 |
| 0 | 0.513286 |
| 161.833 | 0.53018 |
| 0 | 0.505587 |
| 0 | 0.533173 |
| 0 | 0.419042 |
| 205 | 0.804897 |
| 0 | 0.687274 |
| 0 | 0.579518 |
| 0 | 0.436929 |
| 0 | 0.408928 |
| 0 | 0.652227 |
| 0 | 0.610731 |
| 0 | 0.691261 |
| 6 | 0.684637 |
| 5 | 0.801512 |
| 0 | 0.446261 |
| 0 | 0.627198 |
| 97 | 0.545986 |
| 0 | 0.393126 |
| 21 | 0.724201 |
| 10 | 0.420379 |
| 0 | 0.507023 |
| 47 | 0.667049 |
| 30 | 0.556798 |
| 1 | 0.535844 |
| 0 | 0.483629 |
| 0 | 0.5012 |
| 105 | 0.658512 |
| 0 | 0.678928 |
| 0 | 0.708316 |
| 19 | 0.572501 |
| 57 | 1.03507 |
| 0 | 0.784911 |
| 0 | 0.558115 |
| 0 | 0.778313 |
| 213 | 0.992165 |
| 0 | 0.478594 |
| 0 | 0.698304 |
| 0 | 0.444444 |
| 0 | 0.960804 |
| 167 | 0.971905 |
| 0 | 0.614698 |
| 0 | 0.418407 |
| 2 | 0.344413 |
| 0 | 0.464862 |
| 0 | 0.555609 |
| 0 | 0.315328 |
| 0 | 0.656351 |
| 0 | 0.480013 |
| 0 | 0.369685 |
| 0 | 0.373582 |
| 22 | 0.77799 |
| 0 | 0.532459 |
| 0 | 0.448079 |
| 0 | 0.697317 |
| 0 | 0.581288 |
| 0 | 0.841277 |
| 0 | 0.438381 |
| 0 | 0.561817 |
| 72 | 0.58095 |
| 4 | 0.749896 |
| 185 | 0.573611 |
| 0 | 0.671234 |
| 0 | 0.466654 |
| 184 | 0.523248 |
| 0 | 0.438388 |
| 0 | 0.37851 |
| 0 | 0.666949 |
| 0 | 0.731141 |
| 0 | 0.642996 |
| 42 | 0.419299 |
| 0 | 0.579718 |
| 19 | 0.416139 |
| 0 | 0.478445 |
| 0 | 0.492646 |
| 0 | 0.610475 |
| 0 | 0.657823 |
| 118 | 0.487339 |
| 0 | 0.586936 |
| 0 | 0.332052 |
| 0 | 0.593457 |
| 0 | 0.570483 |
| 0 | 0.556606 |
| 0 | 0.46587 |
| 0 | 0.579889 |
| 75 | 0.733371 |
| 0 | 0.530319 |
| 0 | 0.594326 |
| 0 | 0.439938 |
| 3 | 0.735234 |
| 0 | 0.550193 |
| 31 | 0.661913 |
| 0 | 0.808672 |
| 8 | 0.571116 |
| 0 | 0.456787 |
| 0 | 0.757965 |
| 11 | 0.882002 |
| 0 | 0.644485 |
| 0 | 0.736798 |

TKVCR

| (log₁₀[dialysis duration + 1]) | 変化率 |
| --- | --- |
| 0 | 0.494481 |
| 0 | 0.574142 |
| 1.0607 | 0.588471 |
| 0 | 0.500571 |
| 0.90309 | 0.465647 |
| 0 | 0.756224 |
| 0 | 0.750648 |
| 0 | 0.479551 |
| 1.87641 | 0.59425 |
| 0 | 0.893328 |
| 0 | 0.500837 |
| 0 | 0.603638 |
| 2.37639 | 0.70117 |
| 0 | 0.698152 |
| 1.03476 | 0.767467 |
| 0 | 0.562812 |
| 1.44767 | 0.859524 |
| 1.01284 | 0.883616 |
| 0 | 0.725572 |
| 0 | 0.736994 |
| 0 | 0.837218 |
| 0 | 0.818142 |
| 0 | 0.520152 |
| 0 | 0.734597 |
| 0 | 0.933829 |
| 0 | 0.586142 |
| 0 | 0.528263 |
| 0 | 0.626274 |
| 1.17416 | 0.839912 |
| 0 | 0.792157 |
| 0 | 0.631642 |
| 0 | 0.813317 |
| 0 | 0.781682 |
| 0.518514 | 0.833428 |
| 0 | 0.764882 |
| 0 | 0.572269 |
| 0 | 0.481976 |
| 0 | 0.739567 |
| 0 | 0.771493 |
| 0 | 0.559118 |
| 0 | 0.488235 |
| 0 | 0.61084 |
| 0.113943 | 0.574447 |
| 0 | 0.68163 |
| 0.531479 | 0.584781 |
| 0 | 0.746102 |
| 0 | 0.563294 |
| 2.21783 | 0.420662 |
| 0 | 0.531075 |
| 1.59106 | 0.617647 |
| 0 | 0.509816 |
| 1.60206 | 0.6555 |
| 0 | 0.452134 |
| 0 | 0.945797 |
| 0 | 0.537952 |
| 0 | 0.527 |
| 0 | 0.555858 |
| 0 | 0.577286 |
| 0 | 0.563244 |
| 0.778151 | 0.399003 |
| 0 | 0.54137 |
| 0.845098 | 0.729573 |
| 0 | 0.400181 |
| 0 | 0.839955 |
| 0 | 0.670281 |
| 0 | 0.513286 |
| 2.21174 | 0.53018 |
| 0 | 0.505587 |
| 0 | 0.533173 |
| 0 | 0.419042 |
| 2.31387 | 0.804897 |
| 0 | 0.687274 |
| 0 | 0.579518 |
| 0 | 0.436929 |
| 0 | 0.408928 |
| 0 | 0.652227 |
| 0 | 0.610731 |
| 0 | 0.691261 |
| 0.845098 | 0.684637 |
| 0.778151 | 0.801512 |
| 0 | 0.446261 |
| 0 | 0.627198 |
| 1.99123 | 0.545986 |
| 0 | 0.393126 |
| 1.34242 | 0.724201 |
| 1.04139 | 0.420379 |
| 0 | 0.507023 |
| 1.68124 | 0.667049 |
| 1.49136 | 0.556798 |
| 0.30103 | 0.535844 |
| 0 | 0.483629 |
| 0 | 0.5012 |
| 2.02531 | 0.658512 |
| 0 | 0.678928 |
| 0 | 0.708316 |
| 1.30103 | 0.572501 |
| 1.76343 | 1.03507 |
| 0 | 0.784911 |
| 0 | 0.558115 |
| 0 | 0.778313 |
| 2.33041 | 0.992165 |
| 0 | 0.478594 |
| 0 | 0.698304 |
| 0 | 0.444444 |
| 0 | 0.960804 |
| 2.22531 | 0.971905 |
| 0 | 0.614698 |
| 0 | 0.418407 |
| 0.477121 | 0.344413 |
| 0 | 0.464862 |
| 0 | 0.555609 |
| 0 | 0.315328 |
| 0 | 0.656351 |
| 0 | 0.480013 |
| 0 | 0.369685 |
| 0 | 0.373582 |
| 1.36173 | 0.77799 |
| 0 | 0.532459 |
| 0 | 0.448079 |
| 0 | 0.697317 |
| 0 | 0.581288 |
| 0 | 0.841277 |
| 0 | 0.438381 |
| 0 | 0.561817 |
| 1.86332 | 0.58095 |
| 0.69897 | 0.749896 |
| 2.26951 | 0.573611 |
| 0 | 0.671234 |
| 0 | 0.466654 |
| 2.26717 | 0.523248 |
| 0 | 0.438388 |
| 0 | 0.37851 |
| 0 | 0.666949 |
| 0 | 0.731141 |
| 0 | 0.642996 |
| 1.63347 | 0.419299 |
| 0 | 0.579718 |
| 1.30103 | 0.416139 |
| 0 | 0.478445 |
| 0 | 0.492646 |
| 0 | 0.610475 |
| 0 | 0.657823 |
| 2.07555 | 0.487339 |
| 0 | 0.586936 |
| 0 | 0.332052 |
| 0 | 0.593457 |
| 0 | 0.570483 |
| 0 | 0.556606 |
| 0 | 0.46587 |
| 0 | 0.579889 |
| 1.88081 | 0.733371 |
| 0 | 0.530319 |
| 0 | 0.594326 |
| 0 | 0.439938 |
| 0.60206 | 0.735234 |
| 0 | 0.550193 |
| 1.50515 | 0.661913 |
| 0 | 0.808672 |
| 0.954243 | 0.571116 |
| 0 | 0.456787 |
| 0 | 0.757965 |
| 1.07918 | 0.882002 |
| 0 | 0.644485 |
| 0 | 0.736798 |

(B)

TKVCR

| TKV | 変化率 |
| --- | --- |
| 2606.29 | 0.494481 |
| 2080.27 | 0.574142 |
| 2115.09 | 0.588471 |
| 2813.21 | 0.500571 |
| 3581.1 | 0.465647 |
| 3973.98 | 0.756224 |
| 8631.32 | 0.750648 |
| 2379.79 | 0.479551 |
| 3512.53 | 0.59425 |
| 3393.58 | 0.893328 |
| 5579.42 | 0.500837 |
| 5060.6 | 0.603638 |
| 2096.61 | 0.70117 |
| 3765.24 | 0.698152 |
| 5729.52 | 0.767467 |
| 5405 | 0.562812 |
| 1422 | 0.859524 |
| 2002 | 0.883616 |
| 3582 | 0.725572 |
| 2240.6 | 0.736994 |
| 3956.84 | 0.837218 |
| 3600.66 | 0.818142 |
| 4703.32 | 0.520152 |
| 10218.9 | 0.734597 |
| 1653.74 | 0.933829 |
| 2780 | 0.586142 |
| 997.23 | 0.528263 |
| 717.29 | 0.626274 |
| 2826.44 | 0.839912 |
| 2765.71 | 0.792157 |
| 2284.95 | 0.631642 |
| 2745.28 | 0.813317 |
| 1917.67 | 0.781682 |
| 2037.26 | 0.833428 |
| 5018.02 | 0.764882 |
| 1190 | 0.572269 |
| 1154 | 0.481976 |
| 4657 | 0.739567 |
| 2210 | 0.771493 |
| 2705 | 0.559118 |
| 2001 | 0.488235 |
| 7413 | 0.61084 |
| 2333.43 | 0.574447 |
| 3197.07 | 0.68163 |
| 3592.66 | 0.584781 |
| 3804.96 | 0.746102 |
| 2076.75 | 0.563294 |
| 805.92 | 0.420662 |
| 3365.76 | 0.531075 |
| 5024.96 | 0.617647 |
| 3997.4 | 0.509816 |
| 2923.22 | 0.6555 |
| 3108.96 | 0.452134 |
| 3355.69 | 0.945797 |
| 2562.42 | 0.537952 |
| 1634.42 | 0.527 |
| 1589.65 | 0.555858 |
| 4215.19 | 0.577286 |
| 6165.23 | 0.563244 |
| 3414.94 | 0.399003 |
| 3159.63 | 0.54137 |
| 5220.97 | 0.729573 |
| 2566.49 | 0.400181 |
| 3219.66 | 0.839955 |
| 3971.45 | 0.670281 |
| 4049.03 | 0.513286 |
| 1977.92 | 0.53018 |
| 2762.77 | 0.505587 |
| 2220.65 | 0.533173 |
| 2965.24 | 0.419042 |
| 2277.51 | 0.804897 |
| 2592.4 | 0.687274 |
| 1758.8 | 0.579518 |
| 2945.56 | 0.436929 |
| 3729.63 | 0.408928 |
| 3149.09 | 0.652227 |
| 6387.49 | 0.610731 |
| 2224.89 | 0.691261 |
| 2972.92 | 0.684637 |
| 3131.45 | 0.801512 |
| 2366.72 | 0.446261 |
| 5467.06 | 0.627198 |
| 1997.12 | 0.545986 |
| 3335.77 | 0.393126 |
| 1590.25 | 0.724201 |
| 1904.45 | 0.420379 |
| 1516.44 | 0.507023 |
| 2040.87 | 0.667049 |
| 4044.78 | 0.556798 |
| 5140.46 | 0.535844 |
| 2362.14 | 0.483629 |
| 3575.98 | 0.5012 |
| 1476.92 | 0.658512 |
| 4527.09 | 0.678928 |
| 3521.35 | 0.708316 |
| 1692.8 | 0.572501 |
| 1479.76 | 1.03507 |
| 1680.51 | 0.784911 |
| 1878.27 | 0.558115 |
| 2621.9 | 0.778313 |
| 1957.96 | 0.992165 |
| 1193.85 | 0.478594 |
| 2948.43 | 0.698304 |
| 2010.13 | 0.444444 |
| 4562.43 | 0.960804 |
| 2946.42 | 0.971905 |
| 2548.39 | 0.614698 |
| 1705.3 | 0.418407 |
| 5368.91 | 0.344413 |
| 465.45 | 0.464862 |
| 4795.16 | 0.555609 |
| 3535.46 | 0.315328 |
| 4461.29 | 0.656351 |
| 2340.73 | 0.480013 |
| 3853.55 | 0.369685 |
| 5389.18 | 0.373582 |
| 4334.54 | 0.77799 |
| 1711.96 | 0.532459 |
| 5078.55 | 0.448079 |
| 3866.46 | 0.697317 |
| 3491.63 | 0.581288 |
| 5140.91 | 0.841277 |
| 1559.83 | 0.438381 |
| 1995.56 | 0.561817 |
| 2903.16 | 0.58095 |
| 6555.9 | 0.749896 |
| 3316.39 | 0.573611 |
| 2575.36 | 0.671234 |
| 3464.77 | 0.466654 |
| 453.59 | 0.523248 |
| 3174.2 | 0.438388 |
| 2016.54 | 0.37851 |
| 4845.25 | 0.666949 |
| 759.06 | 0.731141 |
| 2152.27 | 0.642996 |
| 2128.91 | 0.419299 |
| 6310.1 | 0.579718 |
| 1242.59 | 0.416139 |
| 2035.95 | 0.478445 |
| 1076.27 | 0.492646 |
| 3424.3 | 0.610475 |
| 2069.63 | 0.657823 |
| 3263.62 | 0.487339 |
| 3497.67 | 0.586936 |
| 2422.12 | 0.332052 |
| 2539.29 | 0.593457 |
| 1980.27 | 0.570483 |
| 797.89 | 0.556606 |
| 617.06 | 0.46587 |
| 2025.94 | 0.579889 |
| 2880.14 | 0.733371 |
| 4416.74 | 0.530319 |
| 3065.89 | 0.594326 |
| 2412.43 | 0.439938 |
| 5430.4 | 0.735234 |
| 1882.52 | 0.550193 |
| 1381.51 | 0.661913 |
| 3073.57 | 0.808672 |
| 1271.16 | 0.571116 |
| 1418.32 | 0.456787 |
| 3443.77 | 0.757965 |
| 5422.72 | 0.882002 |
| 1927.74 | 0.644485 |
| 5316.39 | 0.736798 |

TKVCR

| log₁₀[TKV] | 変化率 |
| --- | --- |
| 3.41602 | 0.494481 |
| 3.31812 | 0.574142 |
| 3.32533 | 0.588471 |
| 3.4492 | 0.500571 |
| 3.55402 | 0.465647 |
| 3.59923 | 0.756224 |
| 3.93608 | 0.750648 |
| 3.37654 | 0.479551 |
| 3.54562 | 0.59425 |
| 3.53066 | 0.893328 |
| 3.74659 | 0.500837 |
| 3.7042 | 0.603638 |
| 3.32152 | 0.70117 |
| 3.57579 | 0.698152 |
| 3.75812 | 0.767467 |
| 3.7328 | 0.562812 |
| 3.1529 | 0.859524 |
| 3.30146 | 0.883616 |
| 3.55413 | 0.725572 |
| 3.35036 | 0.736994 |
| 3.59735 | 0.837218 |
| 3.55638 | 0.818142 |
| 3.6724 | 0.520152 |
| 4.0094 | 0.734597 |
| 3.21847 | 0.933829 |
| 3.44404 | 0.586142 |
| 2.9988 | 0.528263 |
| 2.85569 | 0.626274 |
| 3.45124 | 0.839912 |
| 3.44181 | 0.792157 |
| 3.35888 | 0.631642 |
| 3.43859 | 0.813317 |
| 3.28277 | 0.781682 |
| 3.30905 | 0.833428 |
| 3.70053 | 0.764882 |
| 3.07555 | 0.572269 |
| 3.06221 | 0.481976 |
| 3.66811 | 0.739567 |
| 3.34439 | 0.771493 |
| 3.43217 | 0.559118 |
| 3.30125 | 0.488235 |
| 3.86999 | 0.61084 |
| 3.36799 | 0.574447 |
| 3.50475 | 0.68163 |
| 3.55542 | 0.584781 |
| 3.58035 | 0.746102 |
| 3.31738 | 0.563294 |
| 2.90629 | 0.420662 |
| 3.52708 | 0.531075 |
| 3.70113 | 0.617647 |
| 3.60178 | 0.509816 |
| 3.46586 | 0.6555 |
| 3.49262 | 0.452134 |
| 3.52578 | 0.945797 |
| 3.40865 | 0.537952 |
| 3.21336 | 0.527 |
| 3.2013 | 0.555858 |
| 3.62482 | 0.577286 |
| 3.78995 | 0.563244 |
| 3.53338 | 0.399003 |
| 3.49964 | 0.54137 |
| 3.71775 | 0.729573 |
| 3.40934 | 0.400181 |
| 3.50781 | 0.839955 |
| 3.59895 | 0.670281 |
| 3.60735 | 0.513286 |
| 3.29621 | 0.53018 |
| 3.44134 | 0.505587 |
| 3.34648 | 0.533173 |
| 3.47206 | 0.419042 |
| 3.35746 | 0.804897 |
| 3.4137 | 0.687274 |
| 3.24522 | 0.579518 |
| 3.46917 | 0.436929 |
| 3.57167 | 0.408928 |
| 3.49819 | 0.652227 |
| 3.80533 | 0.610731 |
| 3.34731 | 0.691261 |
| 3.47318 | 0.684637 |
| 3.49575 | 0.801512 |
| 3.37415 | 0.446261 |
| 3.73775 | 0.627198 |
| 3.3004 | 0.545986 |
| 3.5232 | 0.393126 |
| 3.20147 | 0.724201 |
| 3.27977 | 0.420379 |
| 3.18083 | 0.507023 |
| 3.30982 | 0.667049 |
| 3.60689 | 0.556798 |
| 3.711 | 0.535844 |
| 3.37331 | 0.483629 |
| 3.5534 | 0.5012 |
| 3.16936 | 0.658512 |
| 3.65582 | 0.678928 |
| 3.54671 | 0.708316 |
| 3.22861 | 0.572501 |
| 3.17019 | 1.03507 |
| 3.22544 | 0.784911 |
| 3.27376 | 0.558115 |
| 3.41862 | 0.778313 |
| 3.2918 | 0.992165 |
| 3.07695 | 0.478594 |
| 3.46959 | 0.698304 |
| 3.30322 | 0.444444 |
| 3.6592 | 0.960804 |
| 3.46929 | 0.971905 |
| 3.40627 | 0.614698 |
| 3.2318 | 0.418407 |
| 3.72989 | 0.344413 |
| 2.66787 | 0.464862 |
| 3.6808 | 0.555609 |
| 3.54845 | 0.315328 |
| 3.64946 | 0.656351 |
| 3.36935 | 0.480013 |
| 3.58586 | 0.369685 |
| 3.73152 | 0.373582 |
| 3.63694 | 0.77799 |
| 3.23349 | 0.532459 |
| 3.70574 | 0.448079 |
| 3.58731 | 0.697317 |
| 3.54303 | 0.581288 |
| 3.71104 | 0.841277 |
| 3.19308 | 0.438381 |
| 3.30006 | 0.561817 |
| 3.46287 | 0.58095 |
| 3.81663 | 0.749896 |
| 3.52067 | 0.573611 |
| 3.41084 | 0.671234 |
| 3.53967 | 0.466654 |
| 2.65666 | 0.523248 |
| 3.50163 | 0.438388 |
| 3.30461 | 0.37851 |
| 3.68532 | 0.666949 |
| 2.88028 | 0.731141 |
| 3.3329 | 0.642996 |
| 3.32816 | 0.419299 |
| 3.80004 | 0.579718 |
| 3.09433 | 0.416139 |
| 3.30877 | 0.478445 |
| 3.03192 | 0.492646 |
| 3.53457 | 0.610475 |
| 3.31589 | 0.657823 |
| 3.5137 | 0.487339 |
| 3.54378 | 0.586936 |
| 3.3842 | 0.332052 |
| 3.40471 | 0.593457 |
| 3.29672 | 0.570483 |
| 2.90194 | 0.556606 |
| 2.79033 | 0.46587 |
| 3.30663 | 0.579889 |
| 3.45941 | 0.733371 |
| 3.6451 | 0.530319 |
| 3.48656 | 0.594326 |
| 3.38245 | 0.439938 |
| 3.73483 | 0.735234 |
| 3.27474 | 0.550193 |
| 3.14035 | 0.661913 |
| 3.48764 | 0.808672 |
| 3.1042 | 0.571116 |
| 3.15177 | 0.456787 |
| 3.53703 | 0.757965 |
| 3.73422 | 0.882002 |
| 3.28505 | 0.644485 |
| 3.72562 | 0.736798 |

(C)

TKVCR

| age | 変化率 |
| --- | --- |
| 40.3699 | 0.494481 |
| 51.4822 | 0.574142 |
| 62.1288 | 0.588471 |
| 35.7068 | 0.500571 |
| 48.9616 | 0.465647 |
| 62.3397 | 0.756224 |
| 42.7699 | 0.750648 |
| 56.1644 | 0.479551 |
| 60.0301 | 0.59425 |
| 69.8027 | 0.893328 |
| 43.4932 | 0.500837 |
| 60.3534 | 0.603638 |
| 72.6164 | 0.70117 |
| 47.6658 | 0.698152 |
| 46.0164 | 0.767467 |
| 44.6411 | 0.562812 |
| 52.6329 | 0.859524 |
| 53.8548 | 0.883616 |
| 47.0959 | 0.725572 |
| 56.3589 | 0.736994 |
| 38.8137 | 0.837218 |
| 46.3014 | 0.818142 |
| 49.0932 | 0.520152 |
| 47.7699 | 0.734597 |
| 51.937 | 0.933829 |
| 55.5836 | 0.586142 |
| 56.2712 | 0.528263 |
| 13.7699 | 0.626274 |
| 48.9616 | 0.839912 |
| 46.7479 | 0.792157 |
| 56.2493 | 0.631642 |
| 29.7534 | 0.813317 |
| 34.4329 | 0.781682 |
| 49.0959 | 0.833428 |
| 33.2329 | 0.764882 |
| 63.4904 | 0.572269 |
| 60.8219 | 0.481976 |
| 60.1479 | 0.739567 |
| 48.7288 | 0.771493 |
| 38.6986 | 0.559118 |
| 63.8411 | 0.488235 |
| 39.4493 | 0.61084 |
| 51.8384 | 0.574447 |
| 63.3699 | 0.68163 |
| 64.5205 | 0.584781 |
| 43.4 | 0.746102 |
| 51.8849 | 0.563294 |
| 58.9973 | 0.420662 |
| 67.3945 | 0.531075 |
| 61.7205 | 0.617647 |
| 28.1014 | 0.509816 |
| 54.1644 | 0.6555 |
| 48.4164 | 0.452134 |
| 63.7781 | 0.945797 |
| 60.2849 | 0.537952 |
| 63.4932 | 0.527 |
| 59.9616 | 0.555858 |
| 32.0548 | 0.577286 |
| 46.9205 | 0.563244 |
| 47.3699 | 0.399003 |
| 37.0466 | 0.54137 |
| 65.9973 | 0.729573 |
| 54.3781 | 0.400181 |
| 56.5507 | 0.839955 |
| 64.2493 | 0.670281 |
| 43.474 | 0.513286 |
| 69.5452 | 0.53018 |
| 36.7589 | 0.505587 |
| 44.9479 | 0.533173 |
| 45.6877 | 0.419042 |
| 59.274 | 0.804897 |
| 62.3836 | 0.687274 |
| 60.5644 | 0.579518 |
| 46.4329 | 0.436929 |
| 38.2356 | 0.408928 |
| 55.4493 | 0.652227 |
| 48.5123 | 0.610731 |
| 36.5479 | 0.691261 |
| 48.7151 | 0.684637 |
| 63.2904 | 0.801512 |
| 62.8986 | 0.446261 |
| 34.5973 | 0.627198 |
| 58.5342 | 0.545986 |
| 43.7205 | 0.393126 |
| 39.4356 | 0.724201 |
| 49.8521 | 0.420379 |
| 50.9562 | 0.507023 |
| 34.7425 | 0.667049 |
| 62.0466 | 0.556798 |
| 41.2822 | 0.535844 |
| 50.4548 | 0.483629 |
| 46.6795 | 0.5012 |
| 70.126 | 0.658512 |
| 60.4932 | 0.678928 |
| 52.3288 | 0.708316 |
| 61.1644 | 0.572501 |
| 59.4959 | 1.03507 |
| 28.9452 | 0.784911 |
| 54.7808 | 0.558115 |
| 72.5205 | 0.778313 |
| 71.3288 | 0.992165 |
| 56.6219 | 0.478594 |
| 43.1507 | 0.698304 |
| 65.4959 | 0.444444 |
| 48.6603 | 0.960804 |
| 60.8685 | 0.971905 |
| 66.2164 | 0.614698 |
| 67.4192 | 0.418407 |
| 42.5753 | 0.344413 |
| 41.1342 | 0.464862 |
| 58.2438 | 0.555609 |
| 44.2932 | 0.315328 |
| 42.2247 | 0.656351 |
| 25.5096 | 0.480013 |
| 42.4493 | 0.369685 |
| 42.0493 | 0.373582 |
| 45.2822 | 0.77799 |
| 62.4 | 0.532459 |
| 41.3671 | 0.448079 |
| 37.2658 | 0.697317 |
| 61.6 | 0.581288 |
| 56 | 0.841277 |
| 31.8137 | 0.438381 |
| 59.0082 | 0.561817 |
| 61.0932 | 0.58095 |
| 47.9726 | 0.749896 |
| 71.0986 | 0.573611 |
| 46.9342 | 0.671234 |
| 57.0548 | 0.466654 |
| 60.5781 | 0.523248 |
| 47.4466 | 0.438388 |
| 32.9726 | 0.37851 |
| 72.6795 | 0.666949 |
| 67.5562 | 0.731141 |
| 41.4767 | 0.642996 |
| 53.1562 | 0.419299 |
| 45.8274 | 0.579718 |
| 67.326 | 0.416139 |
| 43.5781 | 0.478445 |
| 60.0055 | 0.492646 |
| 50.4301 | 0.610475 |
| 48.8329 | 0.657823 |
| 53.8 | 0.487339 |
| 48.5151 | 0.586936 |
| 25.9589 | 0.332052 |
| 45.7973 | 0.593457 |
| 52.3233 | 0.570483 |
| 63.3288 | 0.556606 |
| 61.2 | 0.46587 |
| 55.9342 | 0.579889 |
| 62.2466 | 0.733371 |
| 42.4986 | 0.530319 |
| 40.4521 | 0.594326 |
| 47.4904 | 0.439938 |
| 52.6438 | 0.735234 |
| 53.611 | 0.550193 |
| 62.526 | 0.661913 |
| 42.2904 | 0.808672 |
| 65.4329 | 0.571116 |
| 42.1562 | 0.456787 |
| 57.9315 | 0.757965 |
| 58.5699 | 0.882002 |
| 60.4301 | 0.644485 |
| 55.137 | 0.736798 |

TKVCR

| age² | 変化率 |
| --- | --- |
| 1629.73 | 0.494481 |
| 2650.42 | 0.574142 |
| 3859.98 | 0.588471 |
| 1274.98 | 0.500571 |
| 2397.24 | 0.465647 |
| 3886.24 | 0.756224 |
| 1829.26 | 0.750648 |
| 3154.44 | 0.479551 |
| 3603.62 | 0.59425 |
| 4872.42 | 0.893328 |
| 1891.65 | 0.500837 |
| 3642.54 | 0.603638 |
| 5273.15 | 0.70117 |
| 2272.02 | 0.698152 |
| 2117.51 | 0.767467 |
| 1992.83 | 0.562812 |
| 2770.22 | 0.859524 |
| 2900.34 | 0.883616 |
| 2218.02 | 0.725572 |
| 3176.33 | 0.736994 |
| 1506.5 | 0.837218 |
| 2143.82 | 0.818142 |
| 2410.14 | 0.520152 |
| 2281.96 | 0.734597 |
| 2697.45 | 0.933829 |
| 3089.53 | 0.586142 |
| 3166.45 | 0.528263 |
| 189.609 | 0.626274 |
| 2397.24 | 0.839912 |
| 2185.37 | 0.792157 |
| 3163.99 | 0.631642 |
| 885.266 | 0.813317 |
| 1185.62 | 0.781682 |
| 2410.41 | 0.833428 |
| 1104.42 | 0.764882 |
| 4031.03 | 0.572269 |
| 3699.31 | 0.481976 |
| 3617.78 | 0.739567 |
| 2374.49 | 0.771493 |
| 1497.58 | 0.559118 |
| 4075.69 | 0.488235 |
| 1556.25 | 0.61084 |
| 2687.22 | 0.574447 |
| 4015.74 | 0.68163 |
| 4162.9 | 0.584781 |
| 1883.56 | 0.746102 |
| 2692.05 | 0.563294 |
| 3480.68 | 0.420662 |
| 4542.02 | 0.531075 |
| 3809.43 | 0.617647 |
| 789.687 | 0.509816 |
| 2933.78 | 0.6555 |
| 2344.15 | 0.452134 |
| 4067.64 | 0.945797 |
| 3634.27 | 0.537952 |
| 4031.38 | 0.527 |
| 3595.4 | 0.555858 |
| 1027.51 | 0.577286 |
| 2201.54 | 0.563244 |
| 2243.9 | 0.399003 |
| 1372.45 | 0.54137 |
| 4355.64 | 0.729573 |
| 2956.98 | 0.400181 |
| 3197.98 | 0.839955 |
| 4127.97 | 0.670281 |
| 1889.99 | 0.513286 |
| 4836.54 | 0.53018 |
| 1351.22 | 0.505587 |
| 2020.32 | 0.533173 |
| 2087.36 | 0.419042 |
| 3513.4 | 0.804897 |
| 3891.71 | 0.687274 |
| 3668.04 | 0.579518 |
| 2156.01 | 0.436929 |
| 1461.96 | 0.408928 |
| 3074.63 | 0.652227 |
| 2353.45 | 0.610731 |
| 1335.75 | 0.691261 |
| 2373.16 | 0.684637 |
| 4005.68 | 0.801512 |
| 3956.24 | 0.446261 |
| 1196.97 | 0.627198 |
| 3426.26 | 0.545986 |
| 1911.49 | 0.393126 |
| 1555.17 | 0.724201 |
| 2485.23 | 0.420379 |
| 2596.53 | 0.507023 |
| 1207.04 | 0.667049 |
| 3849.78 | 0.556798 |
| 1704.22 | 0.535844 |
| 2545.69 | 0.483629 |
| 2178.97 | 0.5012 |
| 4917.66 | 0.658512 |
| 3659.42 | 0.678928 |
| 2738.3 | 0.708316 |
| 3741.08 | 0.572501 |
| 3539.76 | 1.03507 |
| 837.825 | 0.784911 |
| 3000.94 | 0.558115 |
| 5259.23 | 0.778313 |
| 5087.79 | 0.992165 |
| 3206.04 | 0.478594 |
| 1861.98 | 0.698304 |
| 4289.71 | 0.444444 |
| 2367.82 | 0.960804 |
| 3704.97 | 0.971905 |
| 4384.62 | 0.614698 |
| 4545.35 | 0.418407 |
| 1812.66 | 0.344413 |
| 1692.03 | 0.464862 |
| 3392.34 | 0.555609 |
| 1961.88 | 0.315328 |
| 1782.92 | 0.656351 |
| 650.739 | 0.480013 |
| 1801.94 | 0.369685 |
| 1768.14 | 0.373582 |
| 2050.48 | 0.77799 |
| 3893.76 | 0.532459 |
| 1711.24 | 0.448079 |
| 1388.74 | 0.697317 |
| 3794.56 | 0.581288 |
| 3136 | 0.841277 |
| 1012.11 | 0.438381 |
| 3481.97 | 0.561817 |
| 3732.37 | 0.58095 |
| 2301.37 | 0.749896 |
| 5055.02 | 0.573611 |
| 2202.82 | 0.671234 |
| 3255.25 | 0.466654 |
| 3669.7 | 0.523248 |
| 2251.18 | 0.438388 |
| 1087.19 | 0.37851 |
| 5282.3 | 0.666949 |
| 4563.84 | 0.731141 |
| 1720.32 | 0.642996 |
| 2825.58 | 0.419299 |
| 2100.15 | 0.579718 |
| 4532.79 | 0.416139 |
| 1899.05 | 0.478445 |
| 3600.66 | 0.492646 |
| 2543.2 | 0.610475 |
| 2384.65 | 0.657823 |
| 2894.44 | 0.487339 |
| 2353.71 | 0.586936 |
| 673.865 | 0.332052 |
| 2097.39 | 0.593457 |
| 2737.73 | 0.570483 |
| 4010.53 | 0.556606 |
| 3745.44 | 0.46587 |
| 3128.64 | 0.579889 |
| 3874.64 | 0.733371 |
| 1806.13 | 0.530319 |
| 1636.37 | 0.594326 |
| 2255.34 | 0.439938 |
| 2771.37 | 0.735234 |
| 2874.13 | 0.550193 |
| 3909.5 | 0.661913 |
| 1788.48 | 0.808672 |
| 4281.46 | 0.571116 |
| 1777.14 | 0.456787 |
| 3356.06 | 0.757965 |
| 3430.43 | 0.882002 |
| 3651.8 | 0.644485 |
| 3040.09 | 0.736798 |

**Figure S1. Relationship between candidate predictors and TKVCR before and after transformation.**

Scatter plots of dialysis duration (A), pre-transplant TKV (B), and recipient age (C) against the total kidney volume change ratio (TKVCR) in the development set (n=164), shown on the original scale (top panels) and after transformation (bottom panels). Linearity was assessed using restricted cubic splines with four knots, fitted within the multivariable model. No departure from linearity was detected for recipient age (P=0.20) or pre-transplant TKV (P=0.72). For dialysis duration, 72.6% of values were zero (pre-emptive transplantation), which precluded a meaningful spline assessment; the log₁₀(months + 1) transformation was retained to accommodate this distribution. The contribution of the transformations to model fit was small (ΔAIC 1.45). A sensitivity analysis using the untransformed variables gave similar results (development-set R² 0.161; validation-set R² 0.213; area under the curve 0.819).

**Alt text:** Six scatter plots showing total kidney volume change ratio against recipient age, pre-transplant total kidney volume, and dialysis duration, with each variable shown on its original scale and after transformation.

**Abbreviations:** AIC, Akaike information criterion; TKV, total kidney volume; TKVCR, total kidney volume change ratio.

**Table S4. Sensitivity analysis: Effect of the imputation strategy**

|  | **Overall cohort median (primary)** | **Development set median** |
| --- | --- | --- |
| Imputed value, BMI | 21.8535 | 21.8500 |
| Imputed value, SBP (mmHg) | 132.0 | 131.5 |
| Development set R² | 0.1682 | 0.1681 |
| Validation set R² | 0.2549 | 0.2549 |
| Mean absolute error | 0.1215 | 0.1215 |
| Root mean squared error | 0.1539 | 0.1539 |
| Calibration intercept | −0.396 | −0.394 |
| Calibration slope | 1.671 | 1.668 |
| Accuracy (cutoff 0.62) | 0.783 | 0.783 |
| AUC (cutoff 0.62) | 0.850 | 0.850 |

Missing values for BMI (n=1) and SBP (n=10) were imputed using the median of the overall cohort in the primary analysis. In this sensitivity analysis, medians derived from the development set alone were used, and the model was re-estimated in the development set and re-applied to the validation set. The maximum absolute difference between the two sets of predicted values in the validation set was 0.0006, and no patient changed binary class.

**Abbreviations:** AUC, area under the receiver operating characteristic curve; BMI, body mass index; SBP, systolic blood pressure.

**Table S5. Centre-level sample sizes and TKVCR distributions**

| **Set** | **Centre** | **n** | **Mean** | **SD** | **Median** | **Q1–Q3** |
| --- | --- | --- | --- | --- | --- | --- |
| Development | A | 115 | 0.591 | 0.150 | 0.571 | 0.479–0.675 |
| Development | B | 27 | 0.705 | 0.134 | 0.737 | 0.579–0.816 |
| Development | C | 15 | 0.625 | 0.130 | 0.594 | 0.501–0.726 |
| Development | D | 7 | 0.586 | 0.105 | 0.574 | 0.547–0.633 |
| Validation | E | 22 | 0.601 | 0.188 | 0.642 | 0.437–0.715 |
| Validation | F | 14 | 0.624 | 0.172 | 0.653 | 0.543–0.688 |
| Validation | G | 7 | 0.727 | 0.174 | 0.807 | 0.636–0.835 |
| Validation | H | 5 | 0.749 | 0.104 | 0.784 | 0.696–0.813 |
| Validation | I | 5 | 0.709 | 0.214 | 0.671 | 0.605–0.915 |
| Validation | J | 4 | 0.560 | 0.116 | 0.536 | 0.480–0.615 |
| Validation | K | 3 | 0.710 | 0.244 | 0.594 | 0.570–0.792 |

Centres are labelled A to K and are not identified, in the order of decreasing sample size within each set. TKVCR distributions differed between centres (one-way analysis of variance, P=0.012). One centre contributed 115 of the 224 patients (51.3%).

**Abbreviations**: SD, standard deviation; TKVCR, total kidney volume change ratio.

**Table S6. Sensitivity analysis: Binary classification performance at alternative TKVCR cutoff values in the external validation cohort (n=60)**

| **Cutoff** | **Accuracy** | **Sensitivity** | **Specificity** | **PPV** | **NPV** | **Cohen's κ** | **AUC** |
| --- | --- | --- | --- | --- | --- | --- | --- |
| 0.60 | 0.750 | 0.667 | 0.806 | 0.696 | 0.784 | 0.476 | 0.821 |
| 0.6124 (development-set mean) | 0.800 | 0.846 | 0.765 | 0.733 | 0.867 | 0.600 | 0.850 |
| **0.62 (primary)** | **0.783** | **0.846** | **0.735** | **0.710** | **0.862** | **0.569** | **0.850** |
| 0.65 | 0.717 | 0.867 | 0.567 | 0.667 | 0.810 | 0.433 | 0.790 |
| 0.70 | 0.683 | 0.949 | 0.190 | 0.685 | 0.667 | 0.167 | 0.767 |

The primary cutoff of 0.62 corresponded to the mean TKVCR of the overall cohort and was therefore not independent of the validation set. A cutoff derived from the development set alone (0.6124) is shown for comparison. No validation set observation fell between the two values, so the observed classes were identical and classification differed for a single patient. Classification performance varied across thresholds, and all binary analyses should be regarded as exploratory.

**Abbreviations:** AUC, area under the receiver operating characteristic curve; NPV, negative predictive value; PPV, positive predictive value; TKVCR, total kidney volume change ratio.

**Table S7. Performance in the external validation cohort with 95% confidence intervals (n=60)**

| **Measure** | **Estimate** | **95% CI, patient-level bootstrap** | **95% CI, centre-level bootstrap** |
| --- | --- | --- | --- |
| R² (1 − SSE/SST) | 0.255 | 0.078 to 0.377 | −0.046 to 0.338 |
| Mean absolute error | 0.122 | 0.098 to 0.145 | 0.105 to 0.136 |
| Root mean squared error | 0.154 | 0.128 to 0.178 | 0.138 to 0.169 |
| Calibration intercept | −0.396 | −0.748 to −0.038 | −0.761 to 0.077 |
| Calibration slope | 1.671 | 1.111 to 2.222 | 0.957 to 2.288 |
| Accuracy (cutoff 0.62) | 0.783 | 0.683 to 0.883 | 0.737 to 0.854 |
| AUC (cutoff 0.62) | 0.850 | 0.743 to 0.947 | 0.737 to 0.920 |

CIs were obtained by bootstrap resampling with the fitted model held fixed, using 2000 replications and the percentile method. In the patient-level bootstrap, individual patients were resampled with replacement. In the centre-level bootstrap, centres were resampled with replacement and all patients from each selected centre were included, so that within-centre correlation is reflected in the interval; with only seven validation centres, these intervals are themselves imprecise. R² was calculated as 1 − SSE/SST and is not equivalent to the squared correlation between predicted and observed values, which was 0.323.

**Abbreviations:** AUC, area under the receiver operating characteristic curve; CI, confidence interval; MAE, mean absolute error; RMSE, root mean squared error; SSE, sum of squared errors; SST, total sum of squares; TKVCR, total kidney volume change ratio.
